# Genetics of Spontaneous Intracerebral Hemorrhage: Risk and Outcome

**DOI:** 10.3389/fnins.2022.874962

**Published:** 2022-04-11

**Authors:** Hongxiu Guo, Mingfeng You, Jiehong Wu, Anqi Chen, Yan Wan, Xinmei Gu, Senwei Tan, Yating Xu, Quanwei He, Bo Hu

**Affiliations:** Department of Neurology, Union Hospital, Tongji Medical College, Huazhong University of Science and Technology, Wuhan, China

**Keywords:** intracerebral hemorrhage, genetic variation, human genetics, incidence, prognosis

## Abstract

Spontaneous intracerebral hemorrhage (ICH) is a common fatal event without an effective therapy. Of note, some familial aggregation and inherited tendency is found in ICH and heritability estimates indicate that genetic variations contribute substantially to ICH risk and outcome. Thus, identification of genetic variants that affect the occurrence and outcome may be helpful for ICH prevention and therapy. There are several reviews summarizing numerous genetic variants associated with the occurrence of ICH before, but genetic variants contributing to location distribution and outcome have rarely been introduced. Here, we summarize the current knowledge of genetic variants and pay special attention to location distribution and outcome. So far, investigations have reveled variations in *APOE, GPX1, CR1, ITGAV, PRKCH*, and 12q21.1 are associated with lobar ICH (LICH), while *ACE, COL4A2, 1q22, TIMP1, TIMP2, MMP2, MMP9*, and *TNF* are associated with deep ICH (DICH). Moreover, variations in *APOE, VWF*, 17p12, *HP, CFH, IL6ST*, and *COL4A1* are possible genetic contributors to ICH outcome. Furthermore, the prospects for ICH related genetic studies from the bench to the bed were discussed.

## Graphical Abstract

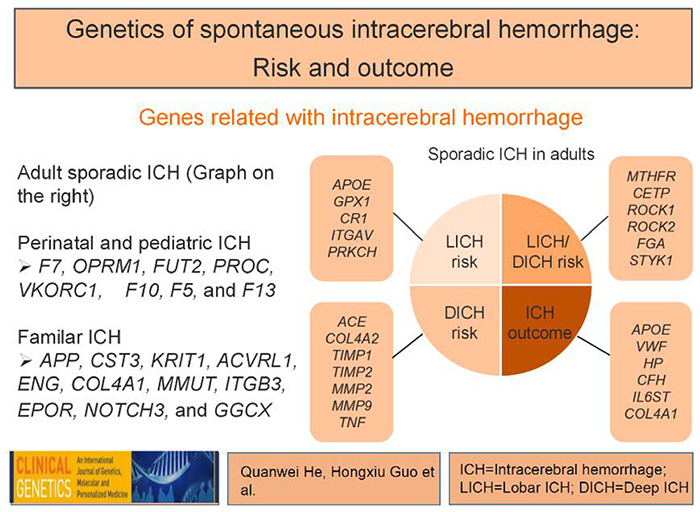

## Introduction

Spontaneous intracerebral hemorrhage (ICH), a form of brain parenchymal hemorrhage caused by non-traumatic events, is one of the most devastating diseases worldwide. It accounts for 10–20% of all strokes, and is associated with 12–39% of long-term functional dependence and a 40% of mortality at 1 month (An et al., 2017). Regarding the location of hemorrhage occurrence within the brain, ICH is routinely classified as deep ICH (DICH) or lobar ICH (LICH). Hypertension is the most important ICH risk factor. Located mainly in deep brain parenchyma, such as the basal ganglia, internal capsule, thalamus, brain stem and cerebellum, hypertensive ICH (HICH) is recognized as the most common type of DICH (Meretoja et al., 2012). Amyloid angiopathy (CAA) is another common cause of ICH, and CAA related hemorrhage (CAA-ICH) accounts for 5–20% of all ICH. Occurring mostly in the lobe of brain, CAA-ICH is accepted as the primary type of LICH.

When ICH attacks, the criminal arteries rupture to form a primary hematoma and immediately expand due to continued bleeding or the mechanical disruption of surrounding vessels. The expanding hematoma mechanically compresses the surrounding brain parenchyma and leads to the primary injury after ICH. Subsequently, the degradation of blood induces oxidative and inflammatory response, contributing to the secondary brain injury (Aronowski and Zhao, 2011). These two processes cause the breakdown of the blood brain barrier (BBB), cerebral edema and neuronal damage, and finally leading to undesirable outcomes of ICH. Unfortunately, there is still no available effective treatment for ICH. Further studies about ICH pathogenesis and pathophysiology are desired to find a novel strategy for ICH prevention and therapy. Identification of genetic variants that affect the occurrence and outcome of ICH may be helpful since some familial aggregation and inherited tendency has been found in ICH. In the study of heritability estimates on ICH, Devan et al. have identified that *APOE* loci and non-*APOE* loci (*CR1* and hypertension-related genes) accounted for some inherited genetic contributions to ICH, with rates at 44, 72, and 51% for risk, admission hematoma volume, and 90-day mortality, respectively (Biffi et al., 2010, 2012; Falcone et al., 2012; Devan et al., 2013).

In this review, a systematic literature search was conducted in PubMed, MEDLINE, Embase, and Web of Science until 01 May 2021, using different combinations of search terms as follows: (intracranial OR cerebral OR intracerebral) AND (hemorrhage OR hematoma OR bleeding) AND (gene OR genetics OR polymorphism OR variant OR mutations OR mutation). Population-based studies including candidate gene studies (case control study, observational/prospective cohort study/meta- analysis) and genome wide association studies (GWAS) with a sample size >50 exploring genetic loci associated with risk and outcome of sporadic ICH in adults were carefully selected and summarized. Moreover, we introduced several genetic determinants focusing on ICH of perinatal/pediatric spectrum and of Mendelian forms.

## Genetic Variants Associated With Sporadic ICH in Adults

### Genes Related to Lobar ICH Risk

#### Apolipoprotein E

Apolipoprotein E (ApoE, encoded by *APOE*) is a polymorphic glycoprotein serving the function of cholesterol transport. *APOE* consists of three common alleles, ε2, ε3, and ε4, and there are six genotypes (ε2/2, ε2/3, ε2/4, ε3/3, ε3/4, and ε4/4). *APOE* may act as a strong genetic cause of ICH, with ε2 and ε4 showing positive, and ε3 showing negative, association with ICH (Talha et al., 2020). Marini et al. (2019) reported that *APOE* ε2 and ε4 alleles appear to affect LICH risk, and Biffi et al. (2010) confirmed the findings and found a novel association between *APOE* ε4 and DICH. Moreover, O’Donnell et al. (2000) found that patients with alleles ε2 and ε4 had a 28% 2-year recurrence rate of ICH, as compared with only 10% in carriers of ε3/ε3 genotype. As for racial differences, genetic studies for ICH occurrence have produced conflicting results. Zhang et al. (2014) reported a significantly higher frequency of *APOE* ε4 allele presented in Asian, European and American patients compared with ICH-free individuals. However, Nie et al. (2019) revealed that both *APOE* ε2 and ε4 alleles acted as risk factors for ICH only in European and American populations, and neither of them had an effect in Asian populations. The pathophysiology underlying the association between *APOE* polymorphisms and ICH is generally accepted to be mediated by CAA. It is supposed that *APOE* ε4 enhances vascular amyloid deposition, while *APOE* ε2 promotes severe CAA responsible for vessel rupture. Moreover, the *APOE* ε4 allele may increase the risk of developing hypertension.

Heritability estimates have identified that *APOE* genetic variations play substantial roles not only in occurrence of ICH, but also in hematoma volume and ICH outcome (Devan et al., 2013). Brouwers et al. (2012a,b) demonstrated that *APOE* ε2 was associated with hematoma expansion and CTA spot sign in patients with LICH. Biffi et al. (2011) found that *APOE*ε2 increased the risk of poor functional outcome (mRS > 2) and mortality at 3 months in LICH. Moreover, functional dependency (Math et al., 2019) as well as poor survival (McCarron et al., 2003) following ICH was observed in patients who possessed the *APOE* ε4. *APOE* ε2 seems to promote the severity and rupture of diseased blood vessels, leading to a larger range of bleeding. *APOE* ε4 appears to amplify neuroinflammatory responses in the setting of ICH, causing increased cerebral edema.

#### Glutathione Peroxidase 1

Glutathione peroxidase 1 (GPX1, encoded by *GPX1*) is an intracellular antioxidant enzyme involved in vascular defense through detoxifying hydrogen. Forgione et al. found that genotypes containing the T allele of C593T polymorphism of GPX1 doubled the risk of LICH compared with CC genotype (Pera et al., 2008). The mechanism is possibly related to amyloid-associated pathology. Deficiency of GPX1 leads to endothelial dysfunction, which could increase susceptibility to oxidative stress-related injury in amyloid-induced vascular damage. Besides, a lack of enzyme GPX1 increases susceptibility to amyloid toxicity in cultured cortical neurons.

#### Complement C3b/C4b Receptor 1

Complement C3b/C4b receptor 1 (CR1, encoded by *CR1*), a member of complement reactivation family, mediates immune responses through binding with the complement component (3b/4b). A genetics of Cerebral Hemorrhage on Anticoagulation (GOCHA) study identified that the *CR1* rs6656401 polymorphism influenced occurrence as well as recurrence of CAA-ICH (Biffi et al., 2012). The *CR1* genetic variability leading to higher risk of LICH is thought to be involved in the clearance of amyloid plaques. Rogers et al. (2006) demonstrated that the CR1 protein was bound to Aβ42 peptide at its C3b ligation adhesion site, while Crehan et al. (2013) found the ability of microglia to phagocytose Aβ was impeded through blocking CR1, resulting in the clearance of Aβ.

#### Integrin Subunit Alpha V

Integrin subunit alpha V (ITGAV, encoded by *ITGAV*) is a glycoprotein belonging to a family of type I transmembrane glycoprotein receptors and mediates a number of cellular functions binding its extracellular ligand to transmit signal. Dardiotis et al. (2017) found that the rs7565633 polymorphism of *ITGAV* was independently associated with LICH risk. One consideration is that ITGAV may affect the integrity of the BBB, as it is implicated in the steadiness of endothelial cell junctions and the tight adhesion of endothelium to the astrocytic perivascular endfeet. Moreover, ITGAV can mediate Aβ-induced toxicity to neurons.

#### Protein Kinase C Eta

Protein kinase C eta (PKC eta, encoded by *PRKCH*) is a serine-threonine kinase essential for cellular signaling transduction and functional regulation of cells, including proliferation, differentiation, and apoptosis. The 1425G/A polymorphism in *PRKCH* enhances the kinase activity. Chen et al. (2012) demonstrated a borderline association between of 1425A and LICH. PKC is involved in the development of atherosclerosis in humans, and the pathophysiology has been proposed to be mediated by inflammation and immunity.

#### 12q21.1

**FIGURE 1:**
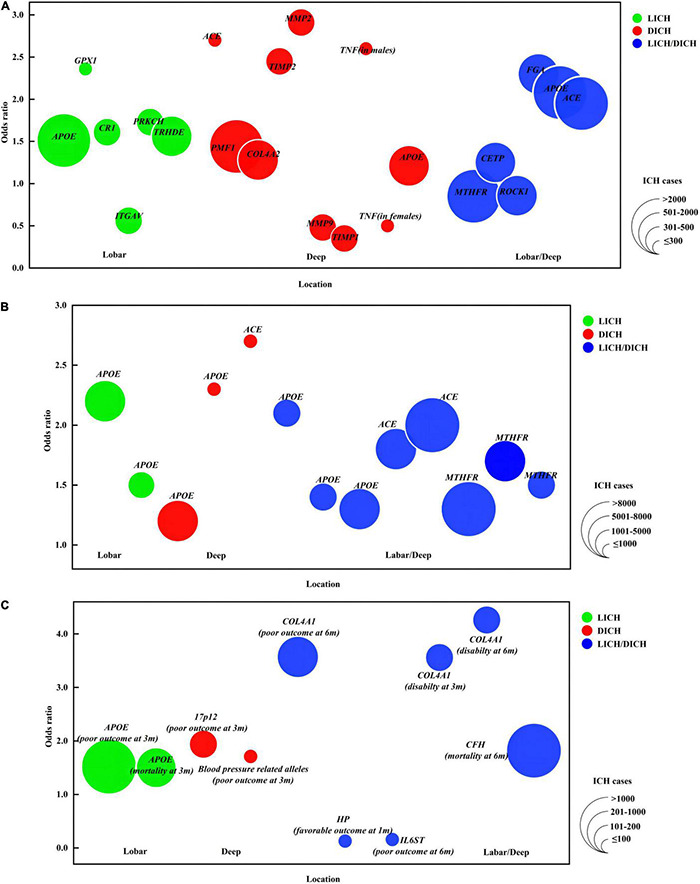
Genetic loci for intracerebral hemorrhage. Colors indicate ICH location. The size of bubbles indicates ICH population. If OR is derived from several variants within one gene or from different genetic models, the OR farthest from 1 is represented here. **(A)** (1) The *X*-axis indicates ICH location. (2) The *Y*-axis provides OR of gene variants associated with ICH risk. **(B)** (1) The *X*-axis indicates ICH location. (2) The *Y*-axis provides OR of gene variants associated with ICH risk replicated in multiple studies/populations. **(C)** (1) The *X*-axis indicates ICH location. (2) The *Y*-axis provides OR of gene variants associated with ICH outcome. *APOE*, apolipoprotein E; *GPX1*, glutathione peroxidase 1; *CR1*, complement C3b/C4b receptor 1; *ITGAV*, integrin subunit alpha V; *PRKCH*, protein kinase C eta; *ACE*, angiotensin I converting enzyme; *COL4A2*, collagen type IV alpha 2 chain; *TIMP*, tissue metalloproteinase inhibitor; *MMP*, matrix metalloproteinase; *TNF*, tumor necrosis factor; *MTHFR*, methylenetetrahydrofolate reductase; *CETP*, cholesteryl ester transfer protein; *ROCK 1*, Rho associated coiled-coil containing protein kinase 1; *FGA*, fibrinogen alpha chain; *HP*, haptoglobin; *IL6ST*, Interleukin 6 cytokine family signal transducer; *COL4A1*, collagen type IV alpha 1 chain; *CFH*, complement factor H; 3/6 m, 3/6 months. Disability (mRS, 3–5); death (mRS, 6); poor outcome (mRS, 3–6); favorable outcome (mRS, 1–2).

A GWAS by Woo et al. (2014) found that several variants on *chr12q21.1*, an intergenic region near the Thyrotropin-releasing hormone-degrading ectoenzyme (TRHDE), were associated with LICH in Europeans, with peak association detected at rs11179580. However, replication in Americans failed to corroborate the positive association (Woo et al., 2014). It is unknown whether the effect estimated in the discovery phase varies across races and how genetic variants on *chr12q21.1* contribute to susceptibility to ICH (Tables 1, 2 and Figures 1A,B).

**TABLE 1:** Genetic variants related to ICH risk in different location (Candidate gene approach).

Ref.	Gene name/Loci and Abb.	Variants	OR (95% CI)	Study population	Cases/Control	Study type	Notes
Pera et al. (2008)	Glutathione peroxidase 1 (*GPX1*)	C593T	2.36 (1.31–4.26)	European	192/192	Candidate gene: CCS	LICH
Biffi et al. (2012)	Complement C3b/C4b receptor 1 (*CR1*)	rs6656401	1.61 (1.19–2.17)	American	369 (89 CAA-ICH)/324	Candidate gene: CCS	CAA-ICH
Dardiotis et al. (2017)	Integrin subunit alpha V (*ITGAV*)	rs7565633	0.56 (0.37–0.86) (Dom)	European	443/572	Candidate gene: CCS	LICH
Chen et al. (2012)	Protein kinase C eta (*PRKCH*)	1425 G/A	1.73 (1.01– 2.9)	Asian	303 (266 DICH/37 LICH)/381	Candidate gene: CCS	LICH
Chen et al. (2008)	Angiotensin I converting enzyme (*ACE)*	*ACE* I/D and A240T (T-D)	2.7 (1.1–6.5)	Asian	217 DICH/283	Candidate gene: CCS	DICH in females
Chen et al. (2015)	Tissue metalloproteinase inhibitor 2 (*TIMP2*)	rs7503607	2.45 (1.37–4.38) (Add)	Asian	396 DICH/376	Candidate gene: CCS	DICH in subjects ≥ 65 years, especially in males
		rs7503726	0.29 (0.10–0.84) (Rec)				DICH in females ≥ 65 years
Chen et al. (2015)	Matrix Metalloproteinase 2 (*MMP2*)	rs2285053	2.91 (1.02–8.31) (Rec)	Asian	396 DICH/376	Candidate gene: CCS	DICH in subjects ≥ 65 years
Ho et al. (2015)	*MMP9*	rs3787268	0.48 (0.27–0.86)	Asian	326 DICH/439	Candidate gene: CCS	DICH in subjects ≥ 65 years
		rs2250889	0.48 (0.27–0.84)				DICH in males < 65 years
Ho et al. (2015)	*TIMP1*	rs4898	0.35 (0.15–0.81)	Asian	326 DICH/439	Candidate gene: CCS	DICH in males ≥ 65 years
Chen et al. (2010)	Tumor necrosis factor (*TNF*)	G-308A	2.6 (1.3– 5.3)	Asian	260 DICH/368	Candidate gene: CCS	DICH in males
		C-863A	0.5 (0.2–0.9)				DICH in females

*CCS, case control study; Dom, dominant; Rec, recessive; Add, additive; LICH, lobar spontaneous intracerebral hemorrhage; DICH, deep spontaneous intracerebral hemorrhage; CAA-ICH, cerebral amyloid angiopathy-related intracerebral hemorrhage; Ref., reference; Abb., Abbreviation; OR, odds ratio.*

**TABLE 2:** Genetic variants related to ICH risk in different location (GWAS).

Ref.	Gene Name/Loci and Abb.	Variants	OR (95% CI)	Study population	Cases/Control	Study type	Notes
Marini et al. (2019)	Apolipoprotein E (*APOE*)	*APOE* ε4	1.51 (1.23–1.85)	American, European	6195 (2305 LICH)/6929	GWAS with meta-analysis	LICH
		*APOE* ε2	1.49 (1.24–1.80)				
Biffi et al. (2010)	*APOE*	*APOE* ε4	1.21 (1.08–1.36)	European	1081 DICH/3657	GWAS with meta-analysis	DICH
Woo et al. (2014)	*12q21.1*	rs11179580	1.56	European, American	1545 (664 LICH/881 DICH)/1481	GWAS	LICH
Woo et al. (2014)	*1q22*	rs2984613	1.44	European, American	3226/3742	GWAS with meta-analysis	DICH
Rannikmäe et al. (2017)	Collagen type IV alpha 2 chain (*COL4A2*)	rs4771674	1.28 (1.13–1.44)	European	1878/2830	GWAS with meta-analysis	DICH

*GWAS, genome-wide association studies; LICH, lobar spontaneous intracerebral hemorrhage; DICH, deep spontaneous intracerebral hemorrhage; Ref., reference; Abb., Abbreviation; OR, odds ratio.*

### Genes Related to Deep ICH Risk

#### Angiotensin I Converting Enzyme

Angiotensin I converting enzyme (ACE, encoded by *ACE*) is a key enzyme of the rennin-angiotensin system, which converts angiotensin I into angiotensin II and inactivates bradykinin. Chen et al. (2008) examined the associations of two polymorphisms (*ACE* A-240T and *ACE* I/D) with ICH and found that haplotype *ACE* T-D polymorphism played minor a role in female DICH risk independent of hypertension, which is consistent with a later study found that *ACE* DD genotype was independently associated with deep hematoma (Kalita et al., 2011). In addition to the effect on ICH occurrence, the *ACE* I/D was reported to influence HICH recurrence as well, with 87.1% of patients deep seated (Misra et al., 2012). Furthermore, Qin et al. (2013) revealed that DD genotype had a greater risk in HICH than that in general ICH, and a lower risk in the subgroup with controls including hypertension than that excluding hypertension, indicating that *ACE* I/D polymorphism and hypertension may have a synergistic effect for ICH. Analyses by race suggested that *ACE* I/D polymorphism was associated with individual susceptibility to ICH in Asians, but not Europeans (Li et al., 2020). The reigning hypothesis of the mechanism is that *ACE* may promote hypertension by enhancing serum ACE levels. Converted by ACE, angiotensin II is involved in the hypertensive development processes, including arteriolar proliferation, smooth muscle cell death and collagen deposition. Moreover, angiotensin II can induce endothelial dysfunction through inflammatory mechanisms. Additionally, chronically high levels of plasma ACE have been shown to increase thickness and stiffness of vascular walls.

#### 1q22

The *chr1q22* locus contains two genes, polyamine-modulated factor 1 (PMF1) gene *PMF1* and solute carrier family 25-member 44 (SLC25A44) gene *SLC25A44*. PMF1 is a nuclear protein required for mitosis and polyamine metabolism. SLC25A44 belongs to the SLC25 family of mitochondrial carrier proteins. Woo et al. (2014) identified several variants related with DICH susceptibility located on *chr1q22*. For the most robust rs2984613, each additional C allele increased the occurrence rate of DICH by 24% (Woo et al., 2014). The variants within *1q22* may alter expression of PMF1 and disrupt polyamine metabolism, which links with cerebrovascular disease through excitatory neurotransmitter receptor regulation and BBB breakdown.

#### Collagen Type IV Alpha 1 Chain and Collagen Type IV Alpha 2 Chain

Collagen type IV alpha 1, 2 chains (COL4A1 and COL4A2, encoded by *COL4A1* and *COL4A2*, respectively), contribute to type IV collagen, a principal component of the vascular basement membrane. Lin et al. (2018) found that the rs544012 AC and rs679505 AA genotypes of *COL4A1* were independently associated with the risk of HICH. Meanwhile, they also demonstrated that rs532625 AA of COL4A1 was associated with higher susceptibility to disability at 3 and 6 months and susceptibility to death/disability at 6 months in HICH patients (Rannikmäe et al., 2015). Rannikmäe et al. (2015) identified three intronic single nucleotide polymorphisms (SNPs) in *COL4A2*, including rs9521732, rs9521733, and rs9515199, contributing to sporadic cases of DICH. Later, in a larger population, nine SNPs in *COL4A2* associated with DICH were identified (Rannikmäe et al., 2017). The accumulation of mutant collagen in vascular endothelial cells and pericytes contributes to basement membrane defects, which weaken the blood vessels and affect vascular function. It has been revealed that *COL4A1* and *COL4A2* missense mutations could change evolutionarily conserved amino acids and attribute to increased intracellular retention of COL4A1 and COL4A2.

#### Matrix Metalloproteinase 2 and Matrix Metalloproteinase 9

Matrix metalloproteinases (MMPs) are a family of zinc/calcium containing endopeptidases and play a crucial role in degrading the extracellular matrix (ECM). Among MMPs, gelatinases, MMP2 (encoded by *MMP2*) and MMP9 (encoded by *MMP9*), are uniquely significant in digesting elastin and collagen of the vascular basement membrane. Chen et al. (2015) and Ho et al. (2015) found that the variants in *MMP2* (rs2285053) and *MMP9* (rs3787268 and rs2250889) promoters could affect DICH risk in view of age and gender, respectively. It is supposed that MMPs contribute to ICH through degrading ECM of vessels. With abnormal presence of MMPs, the deposition of vascular collagen is insufficient, leading to weakness of the vessel walls and the formation of aneurysm. Furthermore, MMPs pathway also plays roles in tissue remodeling and inflammation reactions in ICH.

#### Tissue Inhibitors of Metalloproteinase 1 and Tissue Inhibitors of Metalloproteinase 2

Tissue inhibitors of metalloproteinases (TIMPs) are a family of glycosylated proteins that negatively regulate the proteolytic activity of MMPs. TIMP1 (encoded by *TIMP1*) and TIMP2 (encoded by *TIMP2*) act as the main endogenous inhibitors of MMP9 and MMP2, respectively. Two variants (rs7503607 and rs7503726) of *TIMP2* and one variant (rs4898) of *TIMP1* have been identified as DICH risk factors among subjects of different age and gender (Chen et al., 2015; Ho et al., 2015). TIMPs have been postulated to affect vascular ECM remodeling and breakdown of the BBB by modulating the balance between matrix synthesis and degradation through MMPs inhibition. Moreover, it has been demonstrated that the expression of TIMPs had effects on the development of hypertension, atherosclerosis and intracranial aneurysms.

#### Tumor Necrosis Factor

Tumor necrosis factor (TNF, encoded by *TNF*) is a proinflammatory and immunomodulatory cytokine implicated in the inflammatory process. The *TNF* polymorphisms in its promoter region can lead to an increase of TNF concentration. Chen et al. (2010) reported that the minor alleles −1031C and −308A were positively associated with DICH risk in males, whereas the −863A was inversely associated with DICH risk in females after adjusting for confounding factors such as hypertension, diabetes, etc. TNF can result in the weakening and rupture of the vessel wall through inducing the production of MMPs. Moreover, TNF can reduce the release of tissue plasminogen activator (t-PA) and stimulate the release of plasminogen activator inhibitor type 1 to take the antifibrinolytic effects, which may be involved in early activation of the hemostatic mechanism during a DICH event (Tables 1, 2 and Figures 1A,B).

### Well-Proved Genes Related to Lobar ICH/Deep ICH Risk

#### Methylenetetrahydrofolate Reductase

Methylenetetrahydrofolate reductase (MTHFR, encoded by *MTHFR*) is a ubiquitous cytosolic enzyme playing a key role in homocysteine metabolism. The C677T variant in *MTHFR* can alter the amino acid sequence and enzymatic activity of MTHFR. Several large-scale meta-analyses have shown that *MTHFR* C677T polymorphism was associated with ICH risk (Kang et al., 2013; Zhao and Jiang, 2013; Hu et al., 2016). A prospective study demonstrated that MTHFR TT genotype was a predictor of ICH independent of hypertension (Hultdin et al., 2011). The reduction of enzyme activity caused by *MTHFR* variant leads to high levels of plasma homocysteine, which may cause endothelial dysfunction responsible for atherosclerosis. Other proposed pathogenetic mechanisms are related to alterations of the normal procoagulant-anticoagulant balance and vascular smooth muscle cell proliferation.

#### Cholesteryl Ester Transfer Protein

Cholesteryl ester transfer protein (CETP, encoded by *CETP*) is an important molecule of the lipid metabolism system, mediating exchange of cholesteryl esters and triglycerides between lipoproteins of different density. Anderson et al. (2016) demonstrated an association between twelve *CETP* variants and ICH risk in nearly 6,000 participants, with the strongest association detected at the rs173539 locus. Several variants in *CETP* that result in lower plasma CETP levels and activity have been identified as increasing high-density lipoprotein cholesterol (HDL-C) and total cholesterol (TC) concentrations but lowering low-density lipoprotein cholesterol (LDL-C) (Xu et al., 2016). Dysregulation of lipids influences endothelial function and initiates the formation of early atherosclerotic lesions in arterial vasculature, which can lead to decreased vascular compliance and increased vascular pressure.

#### Rho Associated Coiled-Coil Containing Protein Kinase 1 and Rho Associated Coiled-Coil Containing Protein Kinase 2

Rho associated coiled-coil containing protein kinases (ROCKs), referred to as ROCK1 (encoded by *ROCK1*) and ROCK2 (encoded by *ROCK2*), belong to the AGC family of serine/threonine kinases. Serving as effectors of the RhoA small GTPase, ROCKs are involved in diverse cellular processes, including cell contraction, migration, proliferation and apoptosis. Yang et al. (2018) found that three polymorphisms (rs288980, rs7237677, and rs978906) in *ROCK1* and *ROCK2* were significantly associated with ICH independent of hypertension, which was observed having positive relationship with *ROCK1* polymorphism when following 4,128 subjects. The underlying mechanism for the association is likely a consequence of cerebrovascular atherosclerosis due to dysfunction of endothelial cell and vascular smooth muscle caused by abnormal activation of RhoA/ROCK pathway.

#### Fibrinogen Alpha Chain

Fibrinogen alpha chain (FGA, encoded by *FGA*) is one type of three chains comprising fibrinogen molecules, which is crucial for intravascular thrombus formation and clot viscoelastic properties. Jagiełła et al. (2014) reported that AA genotype of *FGA* Thr312Ala was correlated with a higher prevalence of ICH. The same *FGA* polymorphism has been reported to cause amino-acid substitution and influence clot structure and properties by facilitating factor XIII cross-linking of fibrin fibers (Standeven et al., 2003). It is assumed that *FGA* Thr312Ala variant could increase the risk of ICH involving the coagulation pathway, since the disturbance of blood coagulation contributes to the occurrence of hemorrhage.

#### Serine/Threonine/Tyrosine Kinase 1

Serine/threonine/tyrosine kinase 1 (STYK1, encoded by *STYK1*) is a novel oncogene with kinase domain and is important for regulating intracellular signaling pathways and mediating diverse cellular and developmental processes. An exome-wide association study (EWAS) revealed that rs138533962 polymorphism of *STYK1* had significant associations with ICH occurrence and the prevalence of some intermediate phenotypes, such as hypertension, diabetes mellitus, and hypo-HDL-cholesterolemia (Yamada et al., 2017). Given STYK1’s roles in promoting angiogenesis and changing the vascular morphology during tumor growth, it is deduced that STYK1 may have an effect on the remodeling of blood vessels in the brain (Table 3 and Figures 1A,B).

**TABLE 3:** Well-proved genetic variants related to LICH/DICH risk.

Ref.	Gene name/Locus and Abb.	Variants	OR (95% CI)	Study population	Cases/ Control	Study type (No.)	Notes
Wang et al. (2021)	Methylenetetrahydrofolate reductase (*MTHFR*)	C677T C	0.85	European, American, African, and Asian	3679/9067	Candidate gene: Meta-analysis	ICH
Anderson et al. (2016)	Cholesteryl ester transfer protein (*CETP*)	rs173539	1.25	European, American	1149/1238	GWAS with replication	ICH
Yang et al. (2018)	Rho-associated kinase 1 (*ROCK1*)	rs288980	0.857 (Add)	Asian	607/2443	Candidate gene: CCS	ICH
Jagiełła et al. (2014)	Fibrinogen alpha chain (*FGA*)	Thr312Ala	2.3 (1.1–4.8) (Dom)	European	503/774	Candidate gene: Meta-analysis	ICH
Yamada et al. (2017)	Serine threonine tyrosine kinase 1 (*STYK1*)	rs138533962	111.3 (33.0–694.6)	Asian	673/9158	GWAS	ICH
Chen and Hu (2016)	*APOE*	*APOE* ε4	2.08 (1.57–2.75)	Asian	2018/2143	Candidate gene: Meta-analysis	ICH
Nie et al. (2019)	*APOE*	*APOE* ε2	1.21 (1.07–1.37)	European, American, African, and Asian	1642/5545	Candidate gene: Meta-analysis	ICH in European and American
		*APOE* ε4	1.32 (1.14–1.52)				
Li et al. (2020)	*ACE*	*ACE* I/D	1.95 (1.57–2.43) (Rec); 0.7 (0.6–0.82) (Dom); 0.68 (0.6–0.7) (All)	European, American, African, and Asian	3839/5353	Candidate gene: Meta-analysis	ICH in Asians

*APOE, apolipoprotein E; ACE, angiotensin I converting enzyme; Dom, dominant; Rec, recessive; Add, additive; All, allelic; GWAS, genome-wide association studies; ICH, spontaneous intracerebral hemorrhage; CCS: case control study; Ref., reference; Abb., Abbreviation; OR, odds ratio.*

### Genes Related to Hematoma and Outcome

#### Von Willebrand Factor

Secreted by either endothelial cells or platelets, von Willebrand Factor (vWF, encoded by *VWF*) mediates platelet adhesion and aggregation at the site of vascular injury. Appelboom et al. (2013) found that *VWF* rs216321 polymorphism was significantly associated with relative hematoma growth and approached a borderline association with absolute hematoma growth. It has been reported that the rs216321 variant could decrease the ability of vWF to activate platelets and enhance the ability of vWF to carry factor VIII (Vaidya et al., 2010). Furthermore, combined exposure to hypertension and low levels of vWF largely increased the risk of ICH compared with their separate effects (Johansson et al., 2004), indicating a possible synergistic interaction between them.

#### *17p12* and *22q13*

*17p12* was identified as a genome-wide significant susceptibility locus for hematoma volume of DICH by Marini et al. (2018). Specifically, it was found that rs11655160 polymorphism within the locus was associated with a 6.6-mL decrease in mean ICH volume. Moreover, this same variant was found to be a predictor of lower admission Glasgow coma scale (aGCS) and poor functional outcome (defined as modified Rankin Scale > 2, mRS > 2) at 3 months after ICH (Marini et al., 2018). *22q13* was identified as a locus for ICH volume in LICH, but failed to be replicated (Marini et al., 2018). The biological mechanism underlying the findings is still unknown.

#### Haptoglobin

Haptoglobin (Hp, encoded by *HP*) is an acute phase plasma glycoprotein that promotes the stabilization and clearance of hemoglobin (Hb). In humans, *HP* has two major HP alleles, HP1 and HP2, represented as three genotypes: HP1–1, HP2–1, and HP2–2. Murthy et al. (2015) showed that compared with carriers of HP 1-1 phenotype, patients with HP 2-1/2-2 had a higher occurrence rate of poor functional outcome (mRS > 2) after ICH. Released after ICH, Hb causes the damage of vascular endothelium mediated by free radicals, and leads to disruption of the BBB, resulting in cerebral edema and death of brain parenchymal cells. Hp accelerates the rapid clearance of Hb via CD163 scavenger receptors forming Hp–Hb–CD163 complex on macrophages, exerting its antioxidant activities.

#### Interleukin 6 Cytokine Family Signal Transducer

Interleukin 6 cytokine family signal transducer (IL6ST, encoded by *IL6ST*) performs signal transduction in the processes of immune response, inflammation, the acute phase response and hemopoiesis. Carriers with the minor allele (G) of *IL6ST* rs10940495 were associated with an 84% decrease of having a poor outcome (mRS > 2) at 6 months compared with major allele homozygotes (AA) carriers (El Husseini et al., 2018). Neuroinflammation is thought to cause the secondary brain injury and influence outcomes following ICH, and the formation and homodimerization of the complex of IL-6, IL-6R, and IL6ST can trigger the two main signaling pathways involved in the process of neuroinflammation. Besides, Soluble IL6ST has been found to influence blood pressure in patients with stoke.

#### Complement Factor H

Complement factor H (CFH, encoded by *CFH*) is a primary regulatory component of the alternative pathway, and is crucial for restricting complement action to activating surfaces and regulating complement activation. Appelboom et al. showed that *CFH* rs1061170 polymorphism was independently predictive of mortality at discharge and 6-months, as well as survival duration after ICH onset (Appelboom et al., 2011). In the setting of ICH, thrombin deposits at the hematoma site to activate complement, which then mediates inflammatory reactions such as recruitment of inflammatory cells, release of cytokines and lysis of erythrocytes, contributing to BBB disruption, cerebral edema development and cerebral injury.

### Blood Pressure Related Alleles

Falcone et al. (2013) demonstrated that the genetic risk score (GRS) constructed based on 42 blood pressure related alleles was associated with both baseline hematoma volume and poor clinical outcome (mRS > 2) at 90 days in DICH. Specifically, each additional SD of the score was associated with a 28% increase in mean DICH volume and a 71% increase of poor clinical outcome (Falcone et al., 2013). Patients with more alleles associated with blood pressure possibly suffer from severer hypertensive vasculopathy and show more difficult-to-manage hypertension status, which result in vessel more prone to rupture and hematoma more prone to expansion (Table 4 and Figure 1C).

**TABLE 4:** Genetic variants related to ICH hematoma and outcome.

Ref.	Gene name/Locus and Abb.	Variants	OR (95% CI)	Study population	Sample size	Study type	Notes
Brouwers et al. (2012a)	*APOE*	APOE ε2	2.72 (1.19–6.23)	European	510 (265 LICH)	Candidate gene: PCS	Hematoma expansion after LICH
Brouwers et al. (2012b)	*APOE*	APOE ε2	2.09 (1.05–4.19)	European	371 (196 LICH)	Candidate gene: PCS	Spot sign in LICH
Biffi et al. (2011)	*APOE*	APOE ε2	1.52 (1.25–1.85) for disability; 1.50 (1.23–1.82) for mortality	European, American	2,025 (849 LICH)	GWAS with meta-analysis	Hematoma volume, poor outcome and mortality at 3 m in LICH
Math et al. (2019)	*APOE*	APOE ε4	2.60 (1.25–5.41)	American, European	192	Candidate gene: meta- analysis	Poor outcome
Appelboom et al. (2013)	von Willebrand Factor (*VWF*)	rs216321	–	American	82	Candidate gene: PCS	Relative hematoma growth
Marini et al. (2018)	*17p12*	rs11655160	0.17 for aGCS; 1.94 for disability	European	634 (335 DICH)	GWAS with meta-analysis	Hematoma volume, aGCS and poor outcome at 3 m in DICH
Marini et al. (2018)	*22q13*	rs9614326	–	European	394	GWAS	Hematoma volume
Murthy et al. (2015)	Haptoglobin (*HP*)	HP2-1/2-2	0.13 (0.03–0.71)	American	94	Candidate gene: OCS	Favorable outcome at 30 days
El Husseini et al. (2018)	Interleukin 6 cytokine family signal transducer (*IL6ST*)	rs10940495	0.16 (0.03–0.87)	American	54	Candidate gene: OCS	Poor outcome at 6 m
Appelboom et al. (2011)	Complement factor H (*CFH*)	rs1061170	7.62 (1.40–41.6) for mortality at discharge; 1.822 (1.025–3.239) for mortality at 6 m; 1.822 (1.025–3.239) for survival duration	American, Asian, Hispanic	82	Candidate gene: OCS	Mortality at discharge and 6 m, and survival duration
Xia et al. (2018)	Collagen type IV alpha 2 chain (*COL4A1*)	rs532625	3.557 for disability at 3 m; 4.264 for disability at 6 m; 3.568 for mortality/disability at 6 m	Asian	181 HICH	Candidate gene: PCS	Disability at 3 and 6 m; poor outcome at 6 m
Falcone et al. (2013)	Blood pressure-related alleles	42 SNPs	1.71 (1.05–2.80)	European	323 (135 DICH)	GWAS	Hematoma volume, poor outcome at 3 m in DICH

*APOE, Apolipoprotein E; 3/6 m, 3/6 months; LICH, lobar spontaneous intracerebral hemorrhage; DICH, deep spontaneous intracerebral hemorrhage; HICH, hypertensive intracerebral hemorrhage; SNPs, single nucleotide polymorphisms; aGCS, admission Glasgow coma scale; GWAS, genome-wide association studies; PCS, prospective cohort study; OCS, observational cohort study; Ref., reference; Abb., Abbreviation; OR, odds ratio; No., number of cohorts; mRS, modified Rankin scale. Disability (mRS, 3–5); death (mRS, 6); poor outcome (mRS, 3–6); favorable outcome (mRS, 1–2). Hematoma volume is calculated at ICH presentation.*

## Genetic Variants Associated With Perinatal and Pediatric ICH

Compared with adult ICH, comparatively limited genetic investigations have been conducted in ICH patients of neonates, infants, and children. Coagulation factor VII (FVII) involves in the extrinsic coagulation pathway and functional defects in FVII leading to an autosomal recessive hereditary disorder, congenital factor VII deficiency (FVIID). He et al. (2019) reported a homozygous mutation in the FVII gene (*F7*) splice site (IVS7 + 1G > T) in two cases of neonatal FVIID, and both of them died of severe intracranial hemorrhage. The μ-opioid receptor (OPRM1, encoded by *OPRM1*) is a G protein-coupled receptor involving in cellular signaling pathway. Cheng et al. (2019) revealed that the *OPRM1* A118G polymorphism was associated with ICH in premature infants. The enzyme fucosyltransferase 2 (FUT, encoded by *FUT2*) has the ability to secrete ABH histoblood group antigens into body fluids. Demmert et al. (2015) identified that the *FUT2* G428A was predictive for ICH in a large cohort of very-low-birth weight infants in a recessive genetic model, but the association was not significant any more in the additive genetic model. Protein C (PC, encoded by *PROC*) is a vitamin K-dependent zymogen and exerts anti-coagulant activity through inactivation of Factor V and VIII. Unal et al. (2014) described that a newborn presented with intracranial bleeding had a mutation of T903C in *PROC*. Other genes related to perinatal and pediatric ICH including *VKORC1* (encoding vitamin K epoxide reductase complex subunit 1) (Berber et al., 2018), *F10* (encoding FX) (Herrmann et al., 2005), and *F13* (encoding FXIII) (Göpel et al., 2002; Table 5).

**TABLE 5:** Genetics variants/genes related to perinatal and pediatric ICH and ICH of Mendelian forms.

Ref.	Gene name/Loci and Abb.	Variants	OR (95% CI)	Study population	Cases/Control	Study type	Notes
He et al. (2019)	Factor VII (*F7*)	IVS7 + 1G	–	Asian	2 cases	Case report	P-ICH
Cheng et al. (2019)	μ-opioid receptor (*OPRM1*)	A118G	1.55 (1.00–2.39)	Asian	167/163	Candidate gene: CCS	P-ICH
Demmert et al. (2015)	Fucosyltransferase 2 (*FUT2*)	G428A	1.20 (0.99–1.40)	European	2404	Candidate gene: PCS	P-ICH
Unal et al. (2014)	Protein C (*PROC*)	T903C	–	European	1case	Case report	P-ICH
Berber et al. (2018)	Vitamin K epoxide reductase complex subunit 1 (*VKORC1*)	G1639A	3.63 (1.32–9.94)	European	51/51	Candidate gene: CCS	P-ICH
Herrmann et al. (2005)	Factor X (*F10*)	Gly380Arg	–	European	6 cases	Case report	P-ICH
Göpel et al. (2002)	Factor XIII (*F13*)	Val34Leu	–	European	832	Candidate gene: PCS	P-ICH
Van Broeckhoven et al. (1990)	Amyloid precursor protein (*APP*)	–	–	European	2 families (20 cases)	Case series	HCHWA-D
Palsdottir et al. (1988) and Jensson et al. (1989)	Cystatin C (*CST3*)	–	–	European	8 families (22 cases)	Case series	HCCAA and ICH
Denier et al. (2004)	Krev interaction trapped protein 1 (*KRIT1*)	–	–	European	64 families (202 cases)	Case series	CCM and ICH
Cottin et al. (2007)	Activin receptor-like kinase 1 (*ACVRL1*) and Endoglin (*ENG*)	–	–	European	126 cases	Case series	HHT and ICH
Gould et al. (2006)	Collagen type IV alpha 1 chain (*COL4A1*)	–	–	European	1 family (11 cases)	Case series	BSVD1 and ICH

*CCS, case control study; PCS, prospective cohort study; ICH, intracerebral hemorrhage; P-ICH, perinatal and pediatric ICH; HCHWA-D, human hereditary cerebral hemorrhage with amyloidosis of the Dutch type; HCCAA, hereditary cystatin C amyloid angiopathy; CCM, cerebral cavernous malformations; HHT, hereditary hemorrhagic telangiectasia; BSVD1, brain small vessel disease 1; Ref., reference; Abb., Abbreviation; OR, odds ratio.*

## Genes Related to ICH of Mendelian Forms

In addition to sporadic ICH, ICH with a Mendelian pattern of inheritance caused by monogenic etiologies, such as CAA, cerebral cavernous malformations (CCM), hereditary hemorrhagic telangiectasia (HHT), and brain small vessel disease 1 (BSVD1), have gained great attention all the time. The amyloid precursor protein (APP, encoded by *APP*) is a single-pass transmembrane protein that could generate neurotoxic Aβ peptide in the brain. The *APP* mutation could result in increased deposition of amyloid in the vessel walls and contribute to the most common form of familial CAA, Dutch type, which was associated with increased risk of ICH (Van Broeckhoven et al., 1990). Cystatin C (CST3, encoded by *CST3*) is a cysteine protease inhibitor that regulates cathepsins involving in atherosclerotic lesions of cardiovascular disease. *CST3* mutation could cause production of amyloidogenic protein and contribute to hereditary cystatin C amyloid angiopathy (HCCAA) and subsequent ICH (Palsdottir et al., 1988; Jensson et al., 1989). Krev interaction trapped protein 1 (KRIT1, encoded by *KRIT1*) is a multidomain scaffold protein shown to regulate endothelial cell homeostasis and function. The heterozygous germline mutation in *KRIT1* was described causing CCM, which is a relatively rare cerebrovascular disease linking with seizures and cerebral hemorrhages (Denier et al., 2004). The activin receptor-like kinase 1 (ALK1, encoded by *ACVRL1*) and endoglin (ENG, encoded by *ENG*) are bone morphogenetic protein receptors. Mutations of either one of these two genes (*ACVRL1* and *ENG*) lead to HHT, which appears to cause arteriovenous malformations, thus increase the risk of ICH (Cottin et al., 2007; McDonald et al., 2015). COL4A1 is a constituent of basement membranes of ECM. *COL4A1* mutation could induce disruption of vascular basement membranes and lead to BSVD1, which could increase fragility of cerebral vessels rendering them susceptible to hemorrhage (Gould et al., 2006; Table 5). Other possible genes related to possible monogenic etiologies for ICH including *MMUT* (encoding methylmalonyl-CoA mutase; related to methylmalonic aciduria, MMA), *ITGB3* (encoding glycoprotein IIIa; related to bleeding disorder, Glanzmann thrombasthenia 2 and thrombocytopenia), *EPOR* (encoding erythropoietin receptor; related to familial erythrocytosis), *NOTCH3* (encoding notch receptor 3; related to cerebral arteriopathy with subcortical infarcts and leukoencephalopathy, CADASIL), and *GGCX* (encoding gamma-glutamyl carboxylase; related to deficiency of vitamin K-dependent clotting factors-1, VKCFD1).

## Clinical Implications of Genetic Variants Related to ICH

ICH causes immediate devastation to individuals and there is a lack of available effective treatments at the moment, bringing a heavy burden for family members and society. With the development of personalized medicine and precision medicine, treatment strategies based on genomic data may evolve as a promising approach for disease control. In oncology, genomic biomarkers have gradually been translated into clinical practice (Ginsburg and Kuderer, 2012), which set a paradigm for ICH. Identification of genetic variants associated with the risk, hematoma volume/growth and outcome of ICH can help elucidating pathogenesis and pathophysiology of this disease, and thereby provide guidance for exploring preventive and therapeutic strategies toward individualized and precision ICH management.

Considering different etiologies underlying bleeds in different regions within the brain (LICH was predominantly related to cerebral amyloid angiopathy and DICH was predominantly related to hypertensive and atherosclerotic pathogenesis), some genetic studies identified variants of location-specific to explore genetic mechanisms underlying ICH of lobar and deep location. In this review, we summarized genetic loci associated with increased susceptibility to ICH in terms of location. *APOE, GPX1, CR1, ITGAV, PRKCH*, and *12q21.1* were identified to be associated with LICH, and most of them are involved in CAA-ICH, while *ACE, COL4A2, 1q22, TIMP1, TIMP2, MMP2, MMP9*, and *TNF* were found to be associated with DICH, and mainly by mediating the damage of vascular integrity, and the progression of hypertension and atherosclerosis. We also summarized several well-proved genes related to ICH risk without location restriction, including *MTHFR, CETP, ROCK1, ROCK2, FGA, STYK1*, involved in processes of homocysteine and lipid metabolism, coagulation regulation and inflammation. These findings of genetic risk factors could contribute to ICH prevention by risk assessment and management. For example, individuals with ACE, a high-risk variant relevant to DICH, need to pay more attention to blood pressure control to prevent subsequent ICH. Furthermore, identification of ICH genetic risk factors could be helpful for decision making over chronic anticoagulation. In some clinical situations, such as atrial fibrillation or dilated cardiomyopathy, genetic screening seems plausible to identify subgroup of patients at high ICH risk, thus informing clinicians to better balance the risks and benefits of anticoagulation.

Some genetic loci have been detected as potential prognostic biomarkers of ICH as they may be involved in the primary and secondary injury after ICH, including variants in *APOE, VWF, 17p12, HP, CFH, IL6ST*, and *COL4A1.* These genetic discoveries would be of importance when making treatment strategies. With genetic data, it is possible to identify subjects at high risk of experiencing severe bleedings who are most likely to benefit from early and intensive blood pressure control as well as active hemostatic therapy. Besides, patients who are genetically susceptible to chronic disability and death could be considered for transfer to a neurological intensive care unit and receive more aggressive rehabilitation training.

## Future Directions in ICH Genetics

Due to the small sample size, complex genetic settings and unadvanced genetic study methods, some of the studies reviewed produced conflicting results and the findings remain to be elucidated. Therefore, it is important to enlarge the scale of analyses, evaluate gene–gene and gene–environment interactions, and implement newer genomic technologies and omics methods to acquire more extensive and detailed genetic information concerning ICH. Furthermore, subsequent research is needed to proactively focus on pharmacogenetics, with the expectation of improving personalized and precision management for ICH.

### Gene–Gene and Gene–Environment Interactions

Genetic and environmental factors contribute to ICH development and outcome. Misra et al. (2012) found that *aADDUCIN* (rs4961) GW/WW genotype and *ACE* (rs4646994) DD genotype were both associated with the recurrence of ICH, and homozygous and homo-heterozygous combinations of ACE and *aADDUCIN* variant genotypes (DD + WW and DD/WW + ID/GW) render the patient more vulnerable to recurrent ICH, which means gene-gene interaction may lead to enhanced ICH susceptibility (Misra et al., 2012). Li et al. (2001) found genetic variability of smokers in a study of atherosclerotic peripheral arterial disease (PAD), suggesting that genetic factors may interact with environmental factors. Current studies on gene-gene and gene–environment interactions are relatively few and more comprehensive genetic association studies are required to clarify whether and how genes interact with each other or with environmental factors to influence ICH.

### Whole-Genome/Exome Sequencing and Omics

The molecular genetic studies of ICH were firstly conducted as linkage studies, followed by candidate gene studies and genome wide association studies (GWAS). Having found numerous genetic variants, these alternative studies also have limitations. Except for the inadequate ability to detect rare genetic variants, some variants detected are associated with low probability of phenotypes, and biased results were produced resulting from heterogeneous subjects. Currently, sequencing techniques, referring to whole-genome sequencing and next-generation whole-exome sequencing, are creating novel opportunities. These technologies enable sequencing of continuous reads of DNA, either for coding regions or the entire genome, with the potential to find rare and causal genetic variations contributing to ICH. For further insights into the genetic mechanisms underlying ICH, functional omics methods, including transcriptomics, epigenomics, proteomics and metabolomics, will be worth exploring. Specifically, spatial transcriptomics would be a promising approach to clarify whether location-specific genes were related to different locations in brain tissue. With reasonable combinations of emerging genetic tools and omics, it will be possible to provide a clearer understanding of genetic backgrounds contributing to ICH.

### Pharmacogenomics and Pharmacogenetics

Pharmacogenomics and pharmacogenetics address how genetic variations influence drug responses, helping make decisions on individualized medical therapy for diseases. Actually, evidence has emerged that several genetic variants modulate certain pharmacological drugs which prevent stroke, such as antiplatelet agents and anticoagulants. Cullell et al. (2020) found that several variants in *CYP2C9* (encoding cytochrome P450 2C9) and *VKORC1* (encoding vitamin K epoxide reductase complex subunit 1) associated with acenocoumarol (an oral anticoagulant) maintenance dose were also associated with ICH risk. Moreover, Falcone et al. (2014) demonstrated that *APOE* ε2 and *APOE* ε4 were associated with warfarin related LICH (Falcone et al., 2014). These two examples suggest that reactions of gene polymorphisms in response to drugs should be taken into consideration in the management of ICH. Future studies with larger sample sizes and different ethnic populations are imperative to explore the gene–drug–disease relationships.

Of note, patients with mutations of ICH-related genes have a higher risk for ICH occurrence and suffer from poor outcome after ICH. However, the pathogenic mechanisms remain unknown, and there is a lack of effective treatment. Using *COL4A1* and *COL4A2* mutant mouse models. Jeanne et al. (2015) found that *COL4A1* mutation causes increased intracellular mutant collagen, which leads to vasculature rupture. And they reported treatment of mutant mice with a chemical chaperone approved by US Food and Drug Administration resulted in a decreased collagen intracellular accumulation and a reduced ICH severity (Jeanne et al., 2015). Studies of other ICH-related genes supported by animal models or drug trials involving genetic context and a mechanism-based therapy for ICH are urgently needed in the coming years.

## Discussion

Extensive genetic studies have been performed in the field of ICH risk, but relatively few studies have stressed the location of ICH regarding different etiologies. At the same time, the genetic research on hematoma and outcome of ICH is emerging at high pace. So far investigations have reveled variations in *APOE, GPX1, CR1, ITGAV, PRKCH*, and *12q21.1* are associated with LICH, while *ACE, COL4A2, 1q22, TIMP1, TIMP2, MMP2, MMP9*, and *TNF* are associated with DICH. Moreover, variations in *APOE, VWF, 17p12, HP, CFH, IL6ST*, and *COL4A1* are possible genetic contributors to ICH hematoma and/or outcome. In future, genetic studies are expected to use whole genome/exome sequencing and omics to detect the gene-gene or gene-environment interactions, and to clarify pharmacogenetic implications in ICH. It is believed that with the collaborative efforts of multi-center groups including the International Stroke Genetics Consortium^1^, new avenues for developing appropriate personalized and precision medicine will be opened to fight this devastating disease.

## Author Contributions

HG, MY, and JW did literature review and initial writing. AC, YW, XG, and ST created tables and figures. QH and BH revised the manuscript. YX assembled the final version and subsequent editing of the manuscript. All authors contributed to the study conception and design, read and approved the final manuscript.

## Conflict of Interest

The authors declare that the research was conducted in the absence of any commercial or financial relationships that could be construed as a potential conflict of interest.

## Publisher’s Note

All claims expressed in this article are solely those of the authors and do not necessarily represent those of their affiliated organizations, or those of the publisher, the editors and the reviewers. Any product that may be evaluated in this article, or claim that may be made by its manufacturer, is not guaranteed or endorsed by the publisher.

## Footnotes

1
www.strokegenetics.org
